# Quick Assessment of the Lower Limit of Cerebral Autoregulation Using Transcranial Doppler during Cardiopulmonary Bypass in Cardiac Surgery: A Feasibility Study

**DOI:** 10.31083/j.rcm2406156

**Published:** 2023-05-31

**Authors:** Olivier Desebbe, Etienne Bachelard, Marie Deperdu, Romain Manet, Brenton Alexander, Johanne Beuvelot, Joseph Nloga, Alexandre Joosten, Laurent Gergelé

**Affiliations:** ^1^Department of Anesthesiology and Intensive Care, Ramsay Sante, Sauvegarde Clinic, 69009 Lyon, France; ^2^Department of Anesthesiology and Intensive Care, Lyon Sud University Hospital, Hospices Civils de Lyon, 69495 Pierre Bénite, France; ^3^Lyon Est Medicine Faculty, Claude Bernard Lyon 1 University, 69008 Lyon, France; ^4^Cardiovascular, Metabolic and Nutritional Regulations, Claude Bernard University, 69008 Lyon, France; ^5^Department of Neurosurgery B, P. Wertheimer Hospital, Hospices Civils de Lyon, 69002 Lyon, France; ^6^Department of Anesthesiology, University of California, San Diego, CA 92093, USA; ^7^Department of Cardiac Surgery, Ramsay Health Care, Sauvegarde Clinic, 69009 Lyon, France; ^8^Department of Anesthesiology, Intensive Care & Perioperative Medicine, AP-HP. Paris Saclay University, Paul Brousse Hospital, 94800 Villejuif, France; ^9^Department of Intensive Care, Ramsay Heath Care, Hôpital Privé de la Loire, 42100 Saint Etienne, France

**Keywords:** cerebral autoregulation, cardiopulmonary bypass, transcranial doppler, cardiac surgery

## Abstract

**Background:**

During cardiac surgery, maintaining a mean arterial pressure (MAP) within the range of cerebral autoregulation (CA) may prevent postoperative morbidity. The lower limit of cerebral autoregulation (LLA) can be determined using the mean velocity index (Mx). The standard Mx is averaged over a ten second period (${\mathrm{Mx}}_{\text{10s}}$) while using a two second averaging period (${\mathrm{Mx}}_{\text{2s}}$) is faster and may record more rapid variations in LLA. The objective of this study is to compare a quick determination of LLA (qLLA) using ${\mathrm{Mx}}_{\text{2s}}$ with the reference LLA (rLLA) using ${\mathrm{Mx}}_{\text{10s}}$.

**Methods:**

Single center, retrospective, observational study. Patients undergoing cardiac surgery with cardiopulmonary bypass. From January 2020 to April 2021, perioperative transcranial doppler measuring cerebral artery velocity was placed on cardiac surgery patients in order to correlate with continuous MAP values. Calculation of each patient’s Mx was manually determined after the surgery and qLLA and rLLA were then calculated using a threshold value of Mx >0.4.

**Results:**

55 patients were included. qLLA was found in 78% of the cases versus 47% for rLLA. Despite a –3 mmHg mean bias, limits of agreement were large [–19 mmHg (95% CI: –13; –25), and +13 mmHg (95% CI: +6; +19)]. There was an important interobserver variability (kappa rLLA = 0.46; 95% CI: 0.24–0.66; and Kappa qLLA = 0.36; 95% CI: 0.20–0.52).

**Conclusions:**

Calculation of qLLA is feasible. However, the large limits of agreement and significant interobserver variability prevent any clinical utility or interchangeability with rLLA.

## 1. Introduction 

Cerebral Autoregulation (CA) maintains a constant cerebral blood flow despite changes in blood pressure within an individualized range that is specific to each patient [1]. The mean arterial pressure (MAP) value at which cerebral blood flow (CBF) begins to decrease is called the Lower Limit of Autoregulation (LLA). During cardiopulmonary bypass (CPB) in the context of cardiac surgery, maintaining a MAP below the LLA is associated with an increased risk of postoperative morbidity [2, 3, 4, 5, 6]. The large interindividual variations in LLA make it necessary to use an individualized technique for the correct calculation of each patient’s LLA in order to define the “best” MAP, allowing for appropriate CBF [2].

Using continuous transcranial doppler to measure the CBF alongside an invasive arterial catheter to measure the MAP, the LLA can be determined by a continuous calculation of the correlation between cerebral blood flow velocity ( mean velocity of the mean cerebral artery (MV)) and MAP, also known as the mean velocity index (Mx) [2, 5, 7]. The Mx is a moving Pearson correlation coefficient and approaches the value of 1 when there is a high correlation between MAP and MV (outside of autoregulation) and approaches 0 when the MAP is on the plateau of autoregulation. In order to determine the LLA, a range of MAP values are required to obtain a correlated and uncorrelated relationship with the MV [8]. Classically, the LLA was mainly used within the neuro intensive care unit and was calculated using a large time window (at least five minutes to get the first value of Mx, with a new value each minute) [9]. This large averaging time has pros and cons. Extreme and/or aberrant values are less impactful and can be included in the final calculation. Conversely, if these extreme values are real, they will have a smaller effect on the calculated Mx. Additionally, a minimum of 15–20 minutes for the standard LLA calculation may be too long for cardiac surgery setting. Increasing the recording frequency of paired data (MAP and MV) may allow for a faster assessment of LLA and increase the integration of such values into clinical decisions [10].

The main objectives of our study were to demonstrate the clinical feasibility of a quick determination of LLA (qLLA) throughout a 15-minute period during CPB, and to compare this qLLA with the reference LLA (rLLA), which was calculated throughout the entire CPB period. Secondary objectives were to analyse the interindividual variability of qLLA and rLLA.

## 2. Materials and Methods 

### 2.1 Patients 

This was a prospective and observational study with retrospective analysis. Patients undergoing elective cardiac surgery with CPB and aortic clamping for whom transcranial doppler was used between January 2020 to April 2021 were included. This study protocol received the approval of the Institutional Review Board Ramsay Sante, reference number 00010835. All patients gave written informed consent, were verbally asked if they wanted to refuse data recording and received a letter that gave them the option to recuse themselves later. Patients with history of cerebrovascular disease or significant carotid artery stenosis (greater than 60%) were excluded.

### 2.2 Perioperative Care 

Standard monitoring was used throughout the procedure, including EKG (Philips® healthcare, Amsterdam, The Netherlands), pulse oximetry, depth of anaesthesia monitoring (State/Response Entropy®, GE Healthcare, Chicago, IL, USA), regional cerebral oxygen saturation (INVOS, Medtronic®, Minneapolis, MN, USA), invasive arterial pressure measurement (Seldicath, Prodimed®, Paris, France), and temperature monitoring (Mon-a-Therm, Covidien, Mansfield, MA, USA). Arterial blood pressure was recorded continuously using a radial artery catheter. Anaesthesia and skeletal muscle relaxation were maintained during CPB with propofol, remifentanil, and cisatracurium with the goal of maintaining the depth of anaesthesia monitoring between 40–60. Non-pulsatile CPB (Medtronic® AP 40 oxygenator fusion) was achieved with a non-occlusive centrifugal pump (AP 40, Medtronic, Minneapolis, MN, USA) and patients were kept normothermic (>35 °C). Before CPB, heparin was administered according to the heparin dose response using the hepcon HMS system® (HMS PLUS, Medtronic, Minneapolis, MN, USA) and monitored with activating clotting time (ACT). The CPB flow rate was maintained between 2.0 and 2.6 L⋅$\min^{-1}$⋅$m^{-2}$. MAP was kept between 50 and 90 mmHg, norepinephrine was used if hypotension occured. Acid-based status was measured with an α-stat pH management system. Haemoglobin level was kept above 7 g/dL. Other clinical management of CPB was based on local institutional standards.

### 2.3 Autoregulation Monitoring and Calculation 

**Fig. 1.**
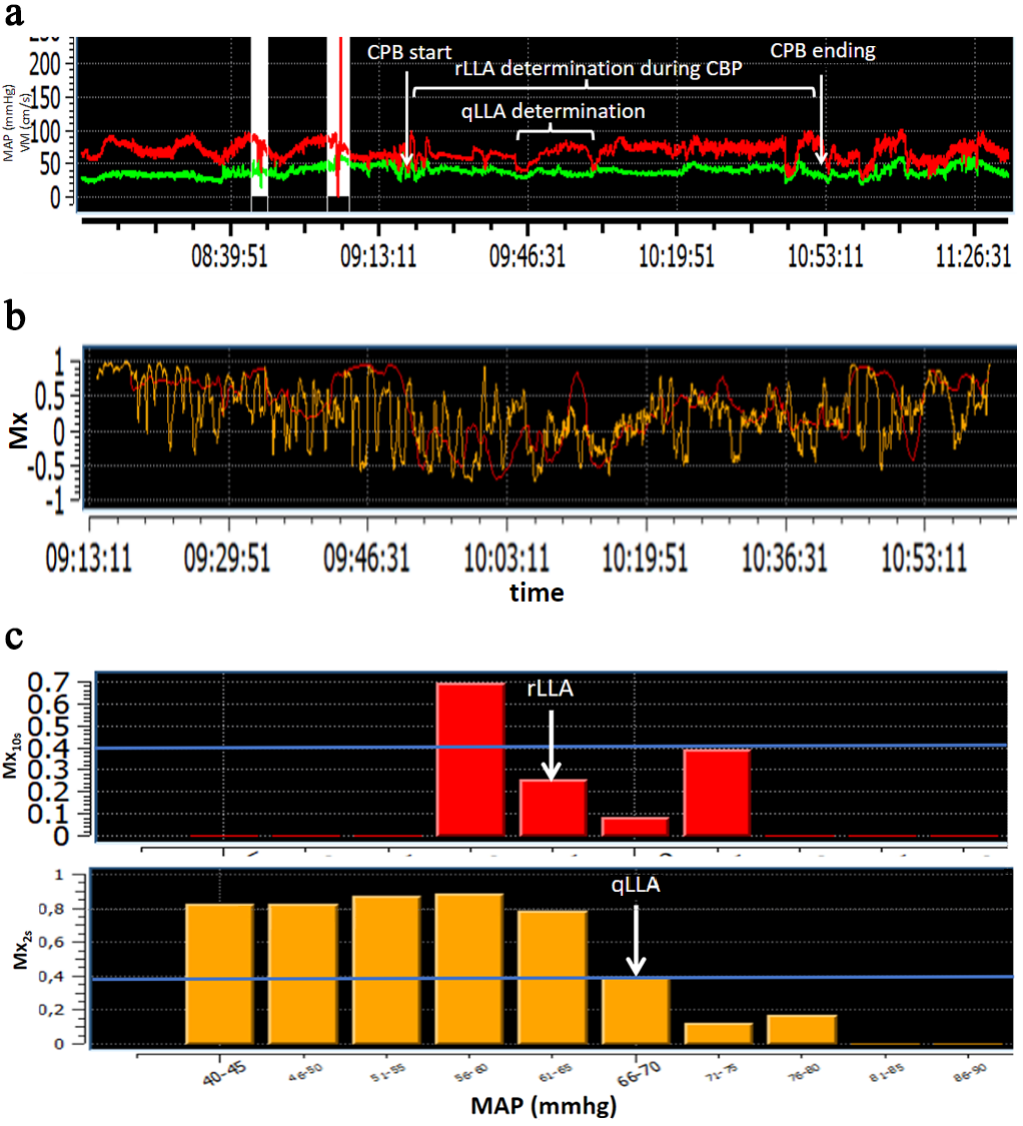
**Determination of qLLA and rLLA using ${\mathrm{Mx}}_{\text{𝟐𝐬}}$ and ${\mathrm{Mx}}_{\text{𝟏𝟎𝐬}}$**. (a) MAP (red tracing) and MV (green tracing) are continuously recorded. The red trace represents the MAP (mmHg), the green line represents the MV (cm/s). Artefacts were manually suppressed before Mx calculation (white zones). (b) ${\mathrm{Mx}}_{\text{2s}}$ (yellow tracing, determination of qLLA) and ${\mathrm{Mx}}_{\text{10s}}$ (red tracing, determination of rLLA) are calculated over time according to the correlation between MAP and MV. (c) ${\mathrm{Mx}}_{\text{2s}}$ (yellow bar) and ${\mathrm{Mx}}_{\text{10s}}$ (red bar) are plotted over MAP range. CPB, cardiopulmonary bypass; qLLA, MAP threshold below the cerebral autoregulation plateau calculated with ${\mathrm{Mx}}_{\text{2s}}$; rLLA, MAP threshold below the cerebral autoregulation plateau calculated with ${\mathrm{Mx}}_{\text{10s}}$; MAP, mean arterial pressure; MV, mean velocity of the mean cerebral artery; ${\mathrm{Mx}}_{\text{2s}}$, calculation of the Mx at 0.5 Hz; ${\mathrm{Mx}}_{\text{10s}}$, Mx calculation at 0.1 Hz; qLLA, quick determination of LLA; rLLA, reference LLA.

MV was continuously measured via transcranial Doppler (WAKIe®, Atys medical, Soucieu-en-Jarrest, France) over the right or left middle cerebral artery with a 2 MHz transducer probe at a depth between 40 and 70 mm. The probe was held in place with a headband. This technology automatically scans all the possible orientations and positions itself, with the subsequent positive flow corresponding to the strongest signal that is manually validated. Additionally, the orientation of the probe automatically readjusts when the signal quality decrease. MV was calculated by the area under the doppler envelope signal. The Doppler envelope is materialized by a white curve that follows the Doppler signal. If the curve did not adequately follow the Doppler signal despite the modifications of the gain, the power, and the width of the sample, the patient was excluded from the analysis. The arterial pressure signal (MAP) was also continuously recorded by the device. Recording frequency of the MV/MAP pair was 1 Hz. Cerebral autoregulation was calculated continuously using the Optimap® software (version 1.3.1, Atys medical, Soucieu en Jarrest, France) present in the doppler device. Optimap® calculates the correlation coefficient between MAP and MV, termed the Mx. Mx determination requires 30 pairs of MAP-MV which are continuously calculated by excluding the oldest of the 30 data pairs and including a new data pair as they are recorded. These new values of Mx (Mxn, Mxn + 1, Mxn + 2) are calculated at a predefined frequency. To calculate qLLA (using Mx average over 2 second period (${\mathrm{Mx}}_{\text{2s}}$)), MV and MAP were collected and averaged at a sampling rate of 0.5 Hz (every two seconds). For the standard calculation of rLLA (using Mx average over 10 second period (${\mathrm{Mx}}_{\text{10s}}$)), data were averaged at a sampling rate of 0.1 Hz (every 10 seconds) (Fig. 1).

**Fig. 2.**
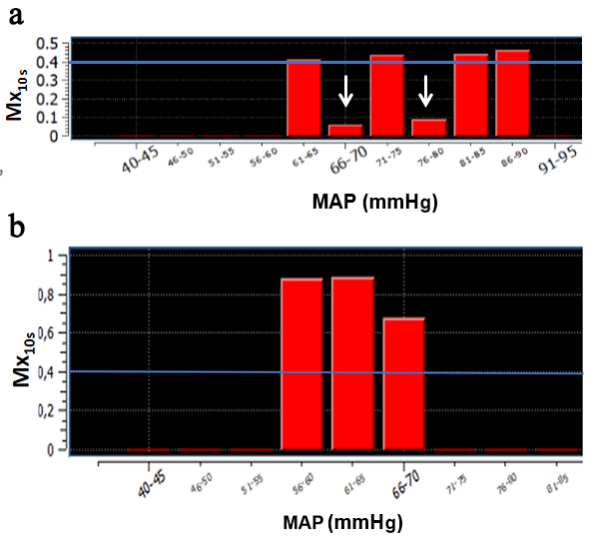
**Examples of LLA indeterminations**. (a) Alternating Mx <0.4 and >0.4, we defined the LLA as Not Calculable (NC). (b) No Mx value was <0.4, this patient was not considered to have a recorded LLA and was defined as No Threshold (NT). MAP, mean arterial pressure; LLA, Low limit of cerebral Autoregulation; Mx, mean velocity index.

The LLA’s were calculated after the surgery. The qLLA was calculated over a period of 15 minutes during CPB and the rLLA was calculated throughout the entire period of CPB. The 15 minutes period was individually chosen by each observer (n = 2) to contain significant variations in MAP (>50%). This implies that the chosen period for calculation potentially differs, as it would be at bedside. The calculation of both qLLA and rLLA was preceded by the manual exclusion of MV and/or MAP artifacts. Mx >0.4 was defined as the threshold of the cerebral autoregulation plateau (LLA) [3, 4]. The LLA was defined as the lowest MAP value with a Mx <0.4 (Fig. 1). If successive Mx’s in the range of MAP did not cross the predefined value of 0.4, the patient was considered to have no recorded LLA and was defined as No Threshold (NT) (Fig. 2). If there were alternating Mx <0.4 and >0.4 in the ranges of MAP, we defined the LLA as Not Calculable (NC) (Fig. 2). In case of different LLA determinations between the two observers, the lead investigator made the final decision.

### 2.4 Statistical Analysis 

The distribution of continuous data was tested for normality using a Shapiro-Wilk test. Normally distributed variables were compared using a student’s *t*-test and expressed as mean ± standard deviation (SD). Variables not normally distributed were compared using a Mann-Whitney U-test and expressed as median (25th–75th percentiles). Discrete data were expressed as numbers and percentages and compared using a Chi square or a Fisher’s exact test when indicated. Interobserver reliability was determined by calculating the Cohen’s kappa index [10].

A Cohen’s kappa value of <0 indicates no agreement, 0–0.20 slight, 0.21–0.40 fair, 0.41–0.60 moderate, 0.61–0.80 substantial, and 0.81–1.00 almost perfect agreement. The agreement between the measurements obtained with qLLA and those obtained with rLLA was assessed using the Bland-Altman method [11]. Significance was set at a 0.05 level. Data were analyzed using MedCalc® Statistical Software version 19.6.4 (MedCalc Software Ltd, Ostend, Belgium; https://www.medcalc.org; 2021).

## 3. Results

**Fig. 3.**
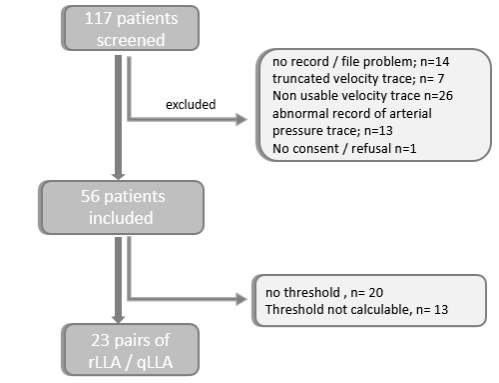
**Flow chart**. Notes: qLLA, MAP threshold below the cerebral autoregulation plateau calculated with ${\mathrm{Mx}}_{\text{2s}}$; rLLA, MAP threshold below the cerebral autoregulation plateau calculated with ${\mathrm{Mx}}_{\text{10s}}$. MAP, mean arterial pressure; qLLA, quick determination of LLA; rLLA, reference LLA.

117 patients were eligible while 55 patients were enrolled (Fig. 3). Patient’s characteristics are shown in the Table 1. qLLA and rLLA were calculable in 78% (n = 43) and 47% (n = 26) of the cases, respectively. ${\mathrm{Mx}}_{\text{2s}}$ was consistently >0.4 in 7% (n = 4) (NT rate) for qLLA determination. NT rate was 35% (n = 20) for rLLA determination with an ${\mathrm{Mx}}_{\text{10s}}$ consistently >0.4 for 18 cases. Non calculable (NC) LLA was found in 15% (n = 8) for qLLA and 16% (n = 9) for rLLA. Average MAP was under the rLLA during 63% of the CPB time (25th–75th: 41–80%). 22 pairs of simultaneous qLLA and rLLA were available. Mean bias between qLLA and rLLA was –3 mmHg (95% CI: –7; +0) and limits of agreement between qLLA and rLLA were –19 mmHg (95% CI: –13; –25), and +13 mmHg (95% CI: +6; +19).

**Table 1.** **Preoperative and per operative characteristics**.

Variables		Mean or median
Age (years)		67 (±9)
BMI (kg/${\mathrm{cm}}^{2}$)		25.6 (±3.9)
Ejection Fraction (%)		61 (60–65)
Male/female gender		73% (n = 40)/27% (n = 15)
Diabetes		18% (n = 10)
Hypertension		65% (n = 36)
Beta-blocker		40% (n = 22)
Hemoglobin (g/dL)		14.4 (13.2–15.1)
Creatinine (µmol/L)		81 (70–90)
Euroscore II (%)		1.1 (0.8–1.3)
CPB duration (min)		66 (48–83)
Type of surgery		
	Coronary artery bypass	42% (n = 23)
	Valvular replacement	35% (n = 19)
	Combined surgery	11% (n = 6)
	Ascending aorta	13% (n = 7)
Aortic cross clamp duration (min)		41 (31–62)
Average MAP (mmHg) during CPB		64 (±6)
Mean CPB pump flow (L/min/$m^{2}$)		2.45 (±0.35)
${\mathrm{SvO}}_{2}$ (%)		80 (±4)
${\mathrm{PaCO}}_{2}$ (mmHg)		48 (47–52)
Hemoglobin (g/dL)		14.4 (13.2–15.1)
qLLA (mmHg)		66 (61–71)
rLLA (mmHg)		66 (66–71)
Minimal and maximal MAP used to calculate ${\mathrm{Mx}}_{\text{2s}}$ (mmHg)		40 (40–51)–80 (75–84)
Minimal and maximal MAP used to calculate ${\mathrm{Mx}}_{\text{10s}}$ (mmHg)		46 (40–51)–80 (70–89)

Notes: Data are presented in mean (± SD) or median (IQR) according to the distribution of the values. BMI, body mass index; Euroscore II, European System for Cardiac Operative Risk Evaluation 2; CPB, cardiopulmonary bypass; ${\mathrm{SvO}}_{2}$, Oxygen venous saturation; ${\mathrm{PaCO}}_{2}$, partial pressure of carbon dioxyde measured by the exhaust delivered by the spectrum; MAP, mean arterial pressure; qLLA, quick determination of LLA; rLLA, reference LLA. qLLA, MAP threshold below the cerebral autoregulation plateau calculated with ${\mathrm{Mx}}_{\text{2s}}$. rLLA, MAP threshold below the cerebral autoregulation plateau calculated with ${\mathrm{Mx}}_{\text{10s}}$.

**Fig. 4.**
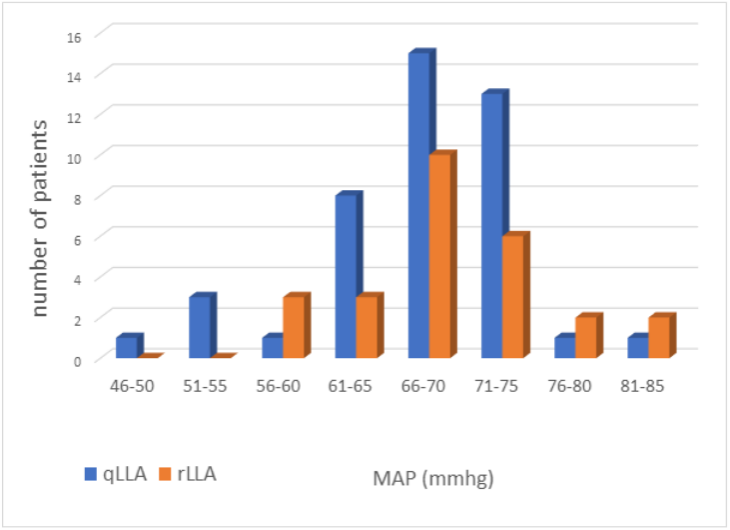
**Individualized qLLA (blue) and rLLA (orange) values**. Notes: qLLA, lower limit of autoregulation determined by the ${\mathrm{Mx}}_{\text{2s}}$ in a short period of 15 minutes; rLLA, lower limit of autoregulation determined by the ${\mathrm{Mx}}_{\text{10s}}$ during the CPB. MAP, mean arterial pressure; CPB, cardiopulmonary bypass; qLLA, quick determination of LLA; rLLA, reference LLA.

Experimenters agreed on identical values of LLA in 48% of the cases for qLLA and in 73% of the cases for rLLA. Coefficient kappa was 0.36 (95% CI: 0.20–0.52), and 0.45 (95% CI: 0.24–0.66), for qLLA and rLLA, respectively (fair and moderate agreement). The dispersion for qLLA and rLLA ranged from 46 to 85 mmHg and 60 to 85 mmHg, respectively (Fig. 4).

## 4. Discussion 

This feasibility study demonstrates that the calculation of cerebral autoregulation with transcranial doppler was possible in 78% of the cases using a quick assessment of LLA on 15 minutes recording compared to only 47% of the cases using the standard analysis on the total CPB recording. The Gaussian distribution of qLLA values (Fig. 4) similar to the distribution of rLLA values suggests that these values are physiological [2].

The large limits of agreement between qLLA and rLLA (±15.8 mmHg) prevent any interchangeability between the two calculations. The poor interobserver agreements and the high frequency of the inability to calculate LLA raises the concern for any bedside applicability of this cerebral autoregulation analysis in this context of observational study. On the other hand, the dispersion of qLLA values was greater, especially in the lower values, which supports the hypothesis of a loss of data by excessive averaging with the reference technique.

Of note, two studies determined the CA with the rLLA for a Mx value of 0.4, but they precised that rLLA was also chosen with the lowest Mx value when the value of 0.4 was not reached [12, 13]. Unfortunately, the authors did not precise the rate of these observations.

Compared to the current use of the longer sampling (${\mathrm{Mx}}_{\text{10s}}$), we hypothesized that a shorter sampling (${\mathrm{Mx}}_{\text{2s}}$) would be more suitable for periods with rapid and intense variations in MAP, such as during cardiac surgery. By analysing these extreme MAP and MV values during a shorter averaging period, we thought that the determination of LLA could be done faster and potentially more effectively. We also considered that if a determination of LLA was able to be performed within a short period of time it may prove valuable for personalizing the subsequent MAP values to avoid dropping below each patient’s LLA, potentially reducing perioperative morbidity.

There are a few potential reasons for our mitigate results. First, the software automatically calculates the LLA according to a predefined period, a predefined sampling of Mx and a predefined threshold value (0.4 for the Mx). As any potential artifacts are incorporated into the Mx calculation, an observer needs to “clean out” these specific periods to ensure appropriate values and then choose individually the 15 minutes period for calculating qLLA. This could explain the large interobserver variability, as mentioned before. Second, the observational design of this study prevented from controlling numerous parameters influencing cerebral autoregulation during cardiopulmonary bypass. We hypothesize that the true individualized LLA is not constant throughout the perioperative period, as the impact of ${\mathrm{PaCO}}_{2}$, ${\mathrm{PaO}}_{2}$, temperature, type of flow, and level of flow likely modify the LLA [14, 15]. Finally, we also excluded patients presenting a significant arterial carotid stenosis. This population could further be assessed because of a potential higher sensitivity of the MV to the MAP.

Moreover, as we did not deliberately change the MAP, ranges of MAP were sometimes narrow which could explain why no threshold was obtained in 35% of the cases for rLLA. Most of these cases presented indeed a ${\mathrm{Mx}}_{\text{10s}}$ >0.4 (Fig. 2). Furthermore, patients with a high ${\mathrm{PaCO}}_{2}$ may have a significantly impaired cerebral autoregulation threshold [16].

This study has limitations. Firstly, there is a limited sample size, due in part to the high rate of unusable MV tracings. We were sometimes transiently faced with a poor Doppler signal despite the initial modifications of the gain, the filtering, and the power of the signal. Hence, the calculation of the mean velocity may have been partially incorrect because of an impropriate signal to noise ratio. Secondly, our institution has only recently started using perioperative transcranial doppler (TCD) technology. As our experience grows, the efficiency and accuracy of the recorded signals will likely improve. During cardiac surgery, an appropriate TCD signal can be challenging to maintain due to patient mobilization and use of electrosurgical unit [17].

Of note, analysis of Mx is one way to calculate cerebral autoregulation and is not considered to be the gold standard despite strong correlation with postoperative complications [2, 4, 5]. Other calculation techniques have been proposed [7], as well as other CBF monitoring techniques, such as cerebral oximetry [18]. The clinical challenge is to find an appropriate method that can continuously analyze the relationship between MAP and CBF despite confounding factors such as hemodilution or ${\mathrm{PaCO}}_{2}$ [16]. This also could explain why there were 35% of NT cases during rLLA assessment (long period of calculation, with a mean CPB time of 66 minutes) and only 7% during qLLA assessment (15 minutes period of calculation). Interestingly, 63% of the aortic clamping period was spent with MAP values lower than the rLLA, which seems correlated to NT cases under the cerebral autoregulation plateau (${\mathrm{Mx}}_{\text{10s}}$ >0.4) during all the CPB. In our study, we retrospectively collected five cases of postoperative delirium and one haemorrhagic stroke. However, in the current practice, we do not assess systematically postoperative cognitive function, which probably led to miss significant neurological insults. Protocols aiming at maintaining a MAP above the LLA have shown conflicting results, with one having a beneficial impact on postoperative delirium [13], and another demonstrating no impact on long term neurologic complications [12].

### Future Research

CPB is a unique state wherein most determinants of cerebral autoregulation (flow, hematocrit, ${\mathrm{PaCO}}_{2}$, temperature, ${\mathrm{PaO}}_{2}$) can be controlled. By keeping these determinants constant and actively modifying the MAP, LLA may be easier to determine. Finally, the Optimap® software continuously delivers a value of Mx, allowing the rapid titration of appropriate MAP. This can potentially improve postoperative neurologic complications and warrants continued research moving forward.

## 5. Conclusions 

Determination of qLLA during CPB is feasible. However, the large limits of agreement between qLLA and rLLA prevent any interchangeability. Additionally, interobserver variation limits bedside applicability for both qLLA and rLLA in a non-controlled environment. Further studies aimed at modifying MAP to actively determine the LLA may limit the impact of confounding factors.

## Data Availability

The datasets used and/or analyzed during the current study are available from the corresponding author on reasonable request.
